# Patients’ Perceptions of Information and Education for Renal Replacement Therapy: An Independent Survey by the European Kidney Patients' Federation on Information and Support on Renal Replacement Therapy

**DOI:** 10.1371/journal.pone.0103914

**Published:** 2014-07-31

**Authors:** Wim Van Biesen, Sabine N. van der Veer, Mark Murphey, Olga Loblova, Simon Davies

**Affiliations:** 1 Renal Division, Ghent University Hospital, Ghent, Belgium; 2 Department of Medical Informatics, Academic Medical Center, Amsterdam, The Netherlands; 3 Irish Kidney Association, Dublin, Ireland; 4 A&R Edelman, London, United Kingdom; 5 Department of Public Policy, Central European University, Budapest, Hungary; 6 Department of Nephrology, University Hospital of North Staffordshire, Stoke on Trent, United Kingdom; Mario Negri Institute for Pharmacological Research and Azienda Ospedaliera Ospedali Riuniti di Bergamo, Italy

## Abstract

**Background:**

Selection of an appropriate renal replacement modality is of utmost importance for patients with end stage renal disease. Previous studies showed provision of information to and free modality choice by patients to be suboptimal. Therefore, the European Kidney Patients’ Federation (CEAPIR) explored European patients’ perceptions regarding information, education and involvement on the modality selection process.

**Methods:**

CEAPIR developed a survey, which was disseminated by the national kidney patient organisations in Europe.

**Results:**

In total, 3867 patients from 36 countries completed the survey. Respondents were either on in-centre haemodialysis (53%) or had a functioning graft (38%) at the time of survey. The majority (78%) evaluated the general information about kidney disease and treatment as helpful, but 39% did not recall being told about alternative treatment options than their current one. Respondents were more often satisfied with information provided on in-centre haemodialysis (90%) and transplantation (87%) than with information provided on peritoneal dialysis (79%) or home haemodialysis (61%), and were more satisfied with information from health care professionals vs other sources such as social media. Most (75%) felt they had been involved in treatment selection, 29% perceived they had no free choice. Involvement in modality selection was associated with enhanced satisfaction with treatment (OR 3.13; 95% CI 2.72–3.60). Many respondents (64%) could not remember receiving education on how to manage their kidney disease in daily life. Perceptions on information seem to differ between countries.

**Conclusions:**

Kidney patients reported to be overall satisfied with the information they received on their disease and treatment, although information seemed mostly to have been focused on one modality. Patients involved in modality selection were more satisfied with their treatment. However, in the perception of the patients, the freedom to choose an alternative modality showed room for improvement.

## Introduction

Clinical practice guidance for treatment of kidney disease worldwide advocate the provision of information and education to patients, as well as patient involvement in the process of selecting a treatment modality [1]–[3]. Timely and adequately informing and educating kidney patients may result in a more balanced modality selection [4]–[6], promote independence and encourage self-management [7], and may be linked to a better prepared start of dialysis [8].

Previous studies suggested that the majority of patients wants to be given information on different treatment options, and that they want to participate in treatment selection [9],[10]. Still, the quality, comprehensibility and completeness of information and education provided to kidney patients seem to be suboptimal at best [11]–[14]. This may partly explain why some patients have little knowledge of their disease as well as a limited awareness of their different treatment options [15], [16]. Also, it has been reported that many kidney patients perceive a lack of choice in whether to start renal replacement therapy (RRT), or in which modality to select [12], [17]. Study results like these raised concerns among the members of the executive committee of the European Kidney Patients’ Federation (CEAPIR).

CEAPIR was founded in 1981 and is a non-profit umbrella organization for 23 national kidney patients’ associations in European countries ranging from Norway to Portugal, and from the United Kingdom to Latvia. CEAPIR’s main aims are to promote the prevention of kidney disease, establish access to treatment for all European kidney patients, and set a European quality standard for treatment of kidney disease. This quality standard states –among other things– that clear information for and education of patients is necessary to fully inform patients’ choices and decisions (www.ceapir.org).

To inform and support a plan of action to improve attainment of this standard across Europe, CEAPIR wanted to explore the current perception of information provision and education amongst patients in Europe, as the scarce available evidence is largely USA based [12],[13],[18].

Therefore, CEAPIR initiated a European project with the aim to explore patients’ perceptions and satisfaction with regard to information and education in general and on different modalities, and their involvement in the modality selection process.

## Methods

### Survey development and dissemination

The executive committee of CEAPIR – consisting of kidney patients and carers– initiated the survey. They framed and selected the preliminary questions in English based on consensus during a face-to face meeting, followed-up by several discussions via e-mail. They started from CEAPIR’s minimal requirement regarding the necessity of “clear and precise *information* and *education* of patients at every stage of the treatment cycle to fully inform patients on their *choices and decisions* on all available treatment options” (www.ceapir.org). The preliminary questions were then circulated to the national patient societies with a request for feedback. This formed the basis for a draft English, electronic survey that was pilot tested among members of the Irish Kidney Association, and revised accordingly.

For dissemination, CEAPIR asked the national patient societies to translate the survey in their local language, and subsequently distribute it among their members. Societies were encouraged to choose a dissemination strategy that matched their organisation’s infrastructure and ongoing activities. Examples of strategies included a paper version of the survey in the society’s periodical magazine (Germany and Poland), sending it via e-mail while combining it with another survey that was already planned on a related topic (United Kingdom), or launching it on a society’s website.

### Data collection and analyses

A professional data management company collected the data from November 2010 to November 2011; they also arranged data entry and storage. Our primary data analysis consisted of descriptive statistics, for which we presented results as valid percentages. In order to investigate the association between treatment decision making (being somewhat or very involved yes/no; having a treatment choice yes/no) and overall satisfaction with received kidney care (being very satisfied yes/no) we calculated odds ratios (OR) and 95% confidence intervals (CI).

We explored the influence of recall bias (i.e., a systematic error due to differences in accuracy or completeness of remembering past experiences) on respondents’ recollection of receiving information or education at the start of RRT we performed a χ^2^ test comparing responses of respondents who had been on their current modality 2 years or less versus more than 2 years. P-values below.05 were considered to indicate a statistically significant difference between these groups.

To address large differences between countries regarding the number of respondents, we performed a separate analysis for countries with more than 20% of respondents (i.e. Poland and Germany) vs other countries using χ^2^ test, with reporting of Odds ratios and 95% confidence intervals.

All statistical analyses were performed using IBM SPSS Statistics 22 by OL, WVB and SVDV.

The protocol and accompanying information for patients of this retrospective study were evaluated by the ethical committee of the Amsterdam Medical Centre (W14_126#14.17.0160), which waived the need for ethical approval. Patient identification data were anonymized for data storage by CEAPIR and data were de-identified before data analysis. The fact that patients completed and send back the questionnaire, after being informed on the purpose and scope of the survey, was considered equivalent to informed consent.

## Results

In total, 3867 patients from 36 different countries responded to the survey. Table 1 displays their characteristics. Germany and Poland each contributed more than 20% of the respondents. The majority of completed questionnaires were received by postal mail through the national societies (58.6%), whereas 17.7, 15.6 and 8.1% were received via e-mail, the CEAPIR website and other (social) media, respectively.

**Table 1:** Respondents’ characteristics (total n = 3867).

Characteristics	Respondents^a)^
Age (years)	
18 to 29	245 (6.7)
30 to 49	990 (27.2)
50 to 69	1828 (50.2)
70 or older	579 (15.9)
Gender	
Male	1944 (53.9)
Country of residence ^b)^	
Germany	1063 (29.9)
Poland	772 (21.7)
Ireland	269 (7.6)
Hungary	187 (5.3)
Portugal	186 (5.2)
Austria	183 (5.1)
Lithuania	136 (3.8)
Finland	120 (3.4)
United Kingdom	114 (3.2)
Italy	93 (2.6)
Current employment status	
Employed	1340 (37.6)
Not employed	992 (27.8)
Retired	1145 (32.1)
Other	91 (2.6)
Time between kidney disease diagnosis and start of first treatment	
Less than 3 months	973 (26.6)
3 to 12 months	548 (15.0)
More than 12 months	2137 (58.4)
Treatment modality at the time of survey completion	
In-centre haemodialysis	1988 (52.6)
Home haemodialysis	91 (2.4)
Peritoneal dialysis (APD, CAPD)	276 (7.3)
Transplanted	1427 (37.7)
Being prepared for dialysis	257 (6.8)
Time on current modality	
Less than 1 year	151 (25.9)
1 or 2 years	190 (32.5)
3 to 5 years	122 (20.9)
More than 5 years	121 (20.7)
Currently listed on transplant waiting pool	
Yes	871 (35.2)
No	1498 (60.6)
Don’t know	101 (4.1)

Abbreviations: APD, automated peritoneal dialysis; CAPD, continuous ambulatory peritoneal dialysis; RRT, renal replacement therapy.

a) Numbers in this column refer to number of respondents (valid %).

b) Top 10 countries with most respondents, together holding 87.8% of all respondents. Other countries were Azerbaijan, Belarus, Belgium, Bosnia and Herzegovina, Bulgaria, Croatia, Cyprus, Denmark, Estonia, France, Georgia, Greece, Iceland, Latvia, Norway, Romania, Russia, San Marino, Slovakia, Slovenia, Spain, Sweden, Switzerland, The Netherlands, Turkey, Ukraine.

### Information on kidney disease and treatment modalities

The majority of respondents (73.8%) reported to have been provided with information on reduced kidney function in the year prior to starting RRT, while 44.9% recalled receiving diet-related information. More than one third (39.3%) did not remember anyone speaking to them about alternative treatment options than their current one or the possibility of changing treatments (Germany = 34.6%, OR 1.03 (95% CI 0.88–1.20) and Poland = 45.9%, 0R 1.65 (95% CI 1.40–1.99) versus other countries). This did not differ between respondents who had been on their current modality ≤2 versus >2 years (χ^2^ = 0.059, p = 0.8).

**Figure 1:**
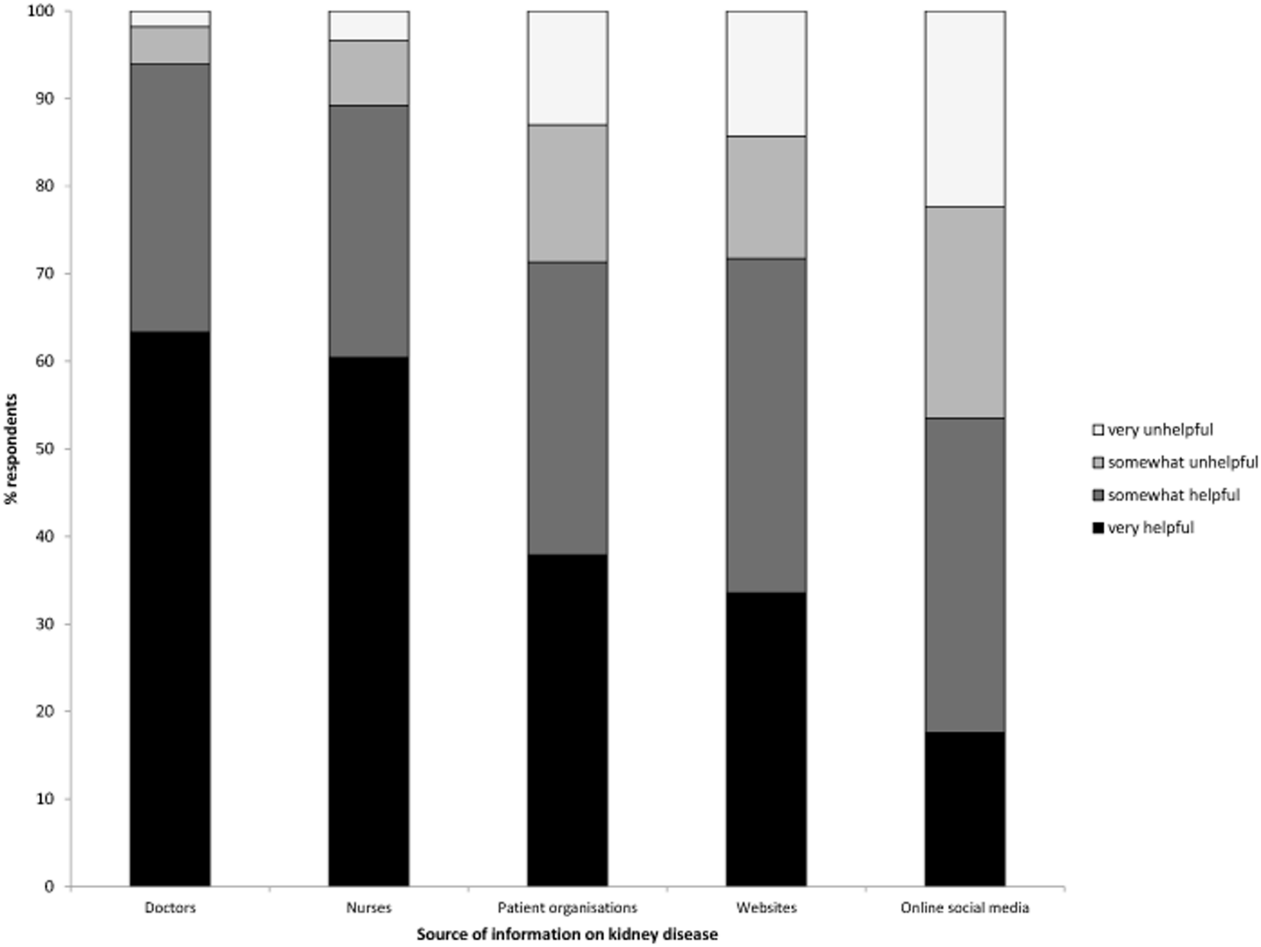
Respondents’ scores on how helpful different information sources on kidney disease have been. Abbreviations: HD, haemodialysis, PD, peritoneal dialysis.

**Figure 2:**
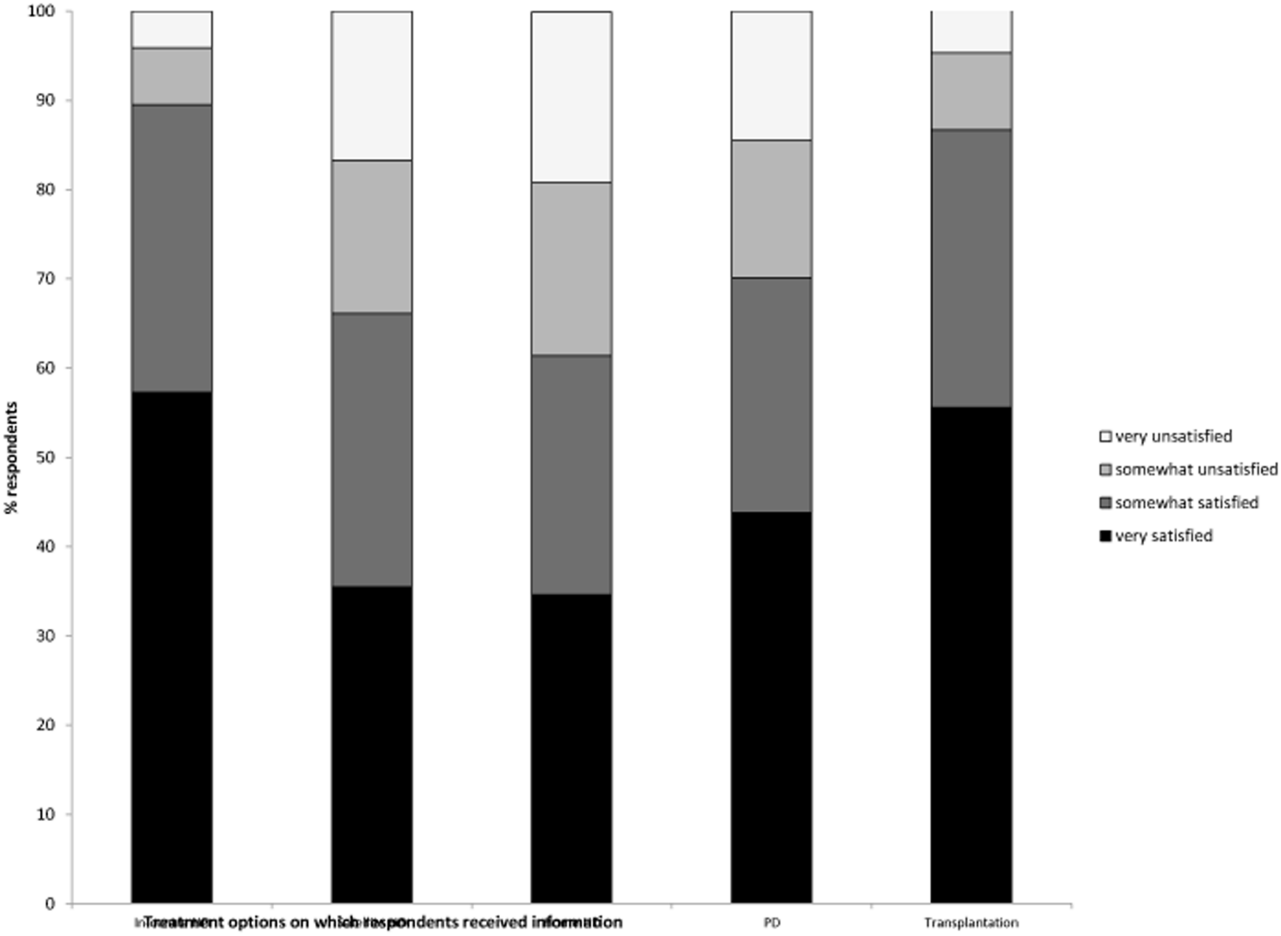
Respondents’ satisfaction with information they had on treatment options.

In general, respondents evaluated the information they got about kidney disease or treatment options as very (46.1%) or somewhat (32.3%) helpful; 11% answered they did not receive or could not remember receiving any information (very useful: Germany = 32.5%, OR 0.43 (95% CI 0.37–0.50) and Poland = 40.9%, OR 0.60 (95% CI 0.52–0.73) versus respondents from other nationalities). For those who said to have received information, the appreciation differed between sources and modalities: Figure 1 shows that health care professionals were more frequently scored as a helpful information source than patient organisations, websites or social media. Furthermore, respondents were more often satisfied with information provided on in-centre haemodialysis and transplantation compared to information on home-based therapies, with which approximately a third of respondents reported to be (very) unsatisfied (Figure 2).

### Treatment decision making

Almost half of respondents indicated that they had been very much (46.7%) involved in the process of selecting a RRT modality (Germany = 46.9%, OR 1.12 (95% CI: 0.96–1.30) and Poland = 31.6%, OR 0.58 (95% CI 0.49–0.70) vs other countries) or somewhat (28.2%) involved. A little more than half (51.2%) perceived they could choose their treatment method (Germany: 57.9%, OR: 1.31; 95% CI 1.12–1.52 and Poland 44.8%, OR 0.77; 95% CI: 0.65–0.91 versus other countries), whereas 48.8% explicitly stated they could not or did not know. Examples of reasons for not having a choice, which respondents provided as free text comments, were that not all modalities were provided in their centre (mostly for home-based therapies), or presence of medical contra-indications. Almost one fourth (22.8%) remembered the medical team suggesting at some time that their social circumstances (e.g., living conditions, work) were a contra-indication for a certain RRT modality (Germany = 24.8%, OR 1.14 (95% CI 0.96–1.35) and Poland = 16.7%, OR 0.69 (95% CI 0.56–0.86) versus other countries).

Almost all respondents were somewhat (31%) or very (63.2%) satisfied with the overall level of their kidney care (very satisfied: Germany = 77.8%, OR 2.51 (95% CI 2.12 to 2.97) and Poland = 42.1%, OR 0.52 (95% CI 0.44 to 0.61) versus other countries). It appeared that participants who felt they had been involved in the modality selection process were more likely to be very satisfied than those who had not been involved (OR 3.13 (95% CI 2.72–3.60); Germany: OR 2.43 (95% CI 1.78–3.31) and Poland: OR 3.30 (95% CI 2.72–3.60). We found a similar association between perceptions of having a choice of treatment and overall satisfaction with care (OR 2.25 (95% CI 1.97–2.56; Germany: OR 1.79 (95% CI 1.34–2.40) and Poland: OR 2.10 (95% CI 1.57–2.81). Time since start dialysis being <2 or >2 years did not influence any of these results.

### Education and support

More than half of the respondents (64.2%) did not receive or could not remember receiving education on how to manage kidney disease in their daily life (Germany = 84.8%, OR 2.50 (95% CI: 2.00–3.00) and Poland = 36.4%, OR 0.25 (95% CI 0.21–0.30) versus other countries). We found no difference between participants who had been on their current modality ≤2 versus >2 years (χ^2^ = 3.3, p = 0.07).

Regarding respondents’ employment status at the time they first started RRT, 27.8% were retired and 3% unemployed. Of the remaining respondents, 22.1% indicated that starting RRT did not affect their employment status. However, 48.4% stopped working, 20.7% reduced their working hours, and 8.7% changed their job. Of respondents who stopped working or changed their job due to their kidney disease, only 22.8% received a training or education to support them with finding a new job.

When asked how satisfied they were with the level of access to a dietician, most participants appeared to be somewhat (34.9%) or very (36.7%) satisfied, while for 9.6% support from a dietician was completely not available (very satisfied: Germany = 60.4%, OR 1.09 (95% CI 0.94–1.27) and Poland = 63.3%, OR 1.24 (95% CI 1.04–1.47) versus other countries). For social workers these percentages were comparable (34.3%, 32.3%, and 12.2%)(very satisfied: Germany = 35.7%, OR 0.56 (95% CI 0.48–0.65) and Poland = 70.1%, OR 2.36 (95% CI 1.98–2.82) versus other countries).

Up to 40.2% of participants did not know whom to address a complaint to, should they ever be dissatisfied with their treatment.

Overall, 20.0% indicated that financial cost was a barrier for optimal treatment for them (Germany = 11.9%, OR 0.78 (95% CI: 0.62–0.98) and Poland = 23.4% (95% CI 1.44–2.17), versus other countries). Patients reporting a financial barrier tended to be less satisfied with their overall care (all respondents OR 0.42 (95% CI 0.35–0.50); Germany: OR 0.30 (95% CI 0.20–0.44) and Poland OR 0.65 (95% CI 0.46–0.92)).

## Discussion

This paper reports the results of a CEAPIR initiated survey that succeeded in involving nearly 4,000 kidney patients from 36 European countries. In general, survey participants reported to be satisfied with the information they received on their disease and treatment, and felt they had been involved in selecting their treatment modality. Moreover, we found being involved to be positively related to overall treatment satisfaction. Nevertheless, more than half of respondents perceived they could not freely choose their modality, and information to facilitate selection of alternative modalities seemed suboptimal, with half of respondents not remembering being presented another modality than their current one. Also education to support the management of kidney disease in patients’ daily and professional lives was perceived suboptimal.

### Modality selection: a free and informed choice?

A substantial part of our respondents did not recall that they were provided with information on alternative modalities. This confirms previous research reporting similar percentages of patients who felt that different therapies were not presented equally for selecting a modality [12], [18]; a systematic review of qualitative studies [13] found that patients did not have the information they wanted on treatment options. Our finding that participants were more satisfied with information on in-centre HD than with home-based therapies is also in line with other studies [5], [12], [18], as was the proportion of respondents perceiving a lack of choice when selecting a modality [12].

There are several potential explanations for the process of modality selection being suboptimal with regard to information and freedom of choice. First, not all potential modalities may be available in a centre, which was also brought forward as a reason by some of our respondents. Morton et al. [13] found that resources often formed the basis of treatment decisions, and that limited access to centre-based dialysis was a consistent reason for choosing home-based therapies. A Danish study suggested that a genuine offer of out-of-centre dialysis would be needed to encourage greater use of modalities other than in-centre haemodialysis [10]. These findings, together with our study results, may warrant future research to investigate to what extent different modalities are actually being offered to kidney patients in European countries. This would inform health policy makers on if and how to stimulate centres to offer all modalities to their patients. It does not necessarily imply that all modalities should be provided in each centre because this may hamper building of experience. Instead, it may be better to, for example, create collaborative structures in which patients can be referred for training on home-based therapies to one central location, and organisational (and financial) barriers to such referrals are addressed.

Secondly, also late referral may jeopardise a free choice of treatment modality. Late referral has been suggested to negatively influence the selection of home based modalities [19], and to contribute to an unbalanced modality mix [20]. More than half of our survey participants recalled the time between the diagnosis of their kidney disease and the start of RRT to be less than three months. Even if we assume that part of these respondents underestimated this time period [12], still for many of them the time would be considered too short for optimal preparation of the patient, e.g., to create an arterio-venous fistula. However, several authors have demonstrated that in centres with dedicated programmes, free modality choice can be offered despite late referral [21]–[23]. Future research may explore the availability of such programmes across Europe.

Thirdly, some health care professionals may refrain from providing information on a particular modality due to their perception of the presence of contra-indications [24], most of which are not absolute [3] and often based on psycho-social concerns [13], [25]. Also in our survey we found medical as well as social contra-indications as perceived reasons for not being offered a certain treatment option. Yet, several studies demonstrated that health care professionals' perceptions may vary widely, depending upon their experience, background, training and personal beliefs [26], [27]. Consequently, part of the kidney patients may be denied a fully free choice based on the presumptions of their healthcare workers. Application of a tool guaranteeing presentation of all available treatment options in a systematic way to patients rather than a tool asking (dichotomous) questions, or the opinion of the educator on what might be of interest to the patient, might help to assure optimal and unprejudiced provision of information.

### Patient involvement in modality selection

We found that a large majority of kidney patients participating in our survey felt that they had been involved in the process of selecting a treatment modality, which appeared to be associated with higher levels of satisfaction with current treatment. Based on their qualitative study, Lee et al. [10] suggested that patients want to participate in choice of modality, but that especially for those on in-centre haemodialysis, selection of alternative modalities was often not facilitated. In general, patients seem increasingly willing to, and asking for, being involved in clinical decisions [28]. At the same time, the model proposed by Flyn et al. [9] made a distinction between patients' preferences regarding receiving information, deliberating that information, and making the final treatment decision. From that study it appeared that all patients want to be informed, but that their preferences for being involved in the deliberation and decision process may vary. Additionally, there is debate on to what extent it is ethical to leave treatment decisions entirely to patients [29]. Nevertheless, health policy makers increasingly advocate patient involvement in decision making, which asks for ways to further optimise this in clinical practice.

One way may be to provide patients with information on experience from other patients, which may strongly influence their decision making [13]. In line with this, Winterbottom *et al.* [*30*] *.* concluded based on a study among students that information provided by patients was considered more influential than when obtained from healthcare professionals. Participants in the CEAPIR survey gave most value to physicians and nurses as a source of information on kidney disease, but unfortunately it was not specifically asked to value information coming from other patients.

Another potential field of focus is the development of tools to engage kidney patients in the decision-making process with their care providers [31], [32]. For renal replacement modality selection, such decision aids should at least mention and explain all available options, taking into account that therapies might be valued differently by different patients for different reasons [33]. Furthermore, the format of clinical practice guidelines should be constructed in a way that stimulates an equal conversation between patient and healthcare worker, rather than just providing a unidirectional flow of information from the clinical perspective [34].

### Information on impact of aspects of disease and treatment on daily living

More than half of the patients in our survey did not remember receiving advise or information on how to manage their kidney disease in their daily life. In the systematic review by Morton et al. [13], the majority of studies indicated that patients and family members were concerned about how to handle practicalities of treatment in their daily life. In most of the included studies, patients judged that provided information was too little focused on minimising impact on daily activities such as working, hobbies, care for children and grandchildren, and too much on length of survival. So, although about 2 out of 3 patients in our survey indicated they satisfied with the availibility of a dietician or a social worker, it is unclear in how far the advice provided by these professionals was really helpful to solve the problems of daily living of attributable to the disease. It is remarkable for example that only one out of four patients in need for a new job indicated they had received assistance for this important aspect of their care.

### Strengths and weaknesses of the study

To our knowledge, this survey is the first to explore the topic of information and education among such a large number of kidney patients. Furthermore, no previous study managed to involve so many different countries, making this survey the first project to explore this topic on a broad international level.

Despite being a relatively small sample from all kidney patients in Europe, our respondents appeared to be representative with regard to treatment modality. For countries participating in the ERA-EDTA registry the percentages of prevalent patients receiving HD in 2011 ranged from 23.8 to 83.6%, 3.7 to 11.3% were treated with PD, and 6.6 to 71.9% had a functioning graft [35]. In our survey, these percentages were 55%, 7.3% and 37.7% for the respective treatment modalities. With regard to the age distribution, our cohort might have been slightly overrepresented in the middle age class (50–69 years) and underrepresented in the age category of 70 years and older. It is unclear from the current survey whether the use of paper survey vs electronic survey only has resulted in more patient participation. However, it is clear that countries were the survey was also available as a paper version are overrepresented in this survey.

We were not able to further investigate the representativeness of our survey participants due to the unsolicited and sometimes indirect nature of the national dissemination strategies, an aspect that might induce selection bias. This prevented us from determining the size and characteristics of the original study population. Apart from impeding calculation of the survey’s response rate, it also hampered a precise comparison between responders and non-responders. In general, younger patients with higher education levels and better health status are more likely to participate in a study like ours than their older, sicker and less educated counterparts [18], [36]. Previous studies suggested that including healthier patients tend to report higher levels of satisfaction with care, which may lead to a too positive picture based on our results. At the same time younger, higher educated participants may have the opposite effect. So, we assume that ultimately the potential selection bias did not significantly influence our results [36], [37].

At the time of initiating the survey, CEAPIR did not intend it to be a research project, but rather an exploration of perceptions among the patients they represent. Although they made an effort to incorporate feedback from national patient organisations and pilot-tested the survey among Irish patients, a strict methodology to ensure unambiguous interpretation of the questions was lacking. On one hand, uniform interpretation may have been further jeopardised by having the survey translated locally without resources to apply a validated method [38]. On the other hand, this most likely decreased selection bias, which in turn increased the representativeness of the study. Also, the survey captured patient perceptions rather than actual practices of information provision and education across Europe. While our results may provide valuable pointers for selecting areas for further research, we cannot use them to determine whether information or education was not provided, or whether it was provided and patients did not recognise it as such or did not remember it. Even though our analyses suggested no effect of recall bias on our findings, these limitations warrant caution when drawing conclusions based on our study results.

### Future research

Based on our study findings, we suggest that it is worthwhile to further investigate the extent to which dialysis centres in Europe offer all modalities to their patients. Additionally, future research may explore the availability of dedicated vascular access programmes in European countries, as well as their effect on the freedom of modality choice for European kidney patients.

CEAPIR delegated the translation and dissemination of the survey to national patient organisations. We assume that having the survey translated in patients' own language greatly increased the number of respondents. However, future initiatives should try to formalise translation by allocating resources to facilitate the use of accepted methods. This will contribute to the methodological rigour of survey-based studies, and improve interpretation of the results. Interestingly, countries that choose to distribute the survey on paper managed to involve a large number of patients. Although we cannot calculate actual response rates, we hypothesize that using paper-based questionnaires may be a way of increasing response and decreasing selection bias in patient populations that are relatively old and with a high disease burden. Further research on this topic is of high relevance. Similar future initiatives should therefore consider to provide surveys on paper in addition to electronic formats.

An analysis of the data separate for Germany and Poland (the two largest contributors) versus other nationalities, demonstrated that for some topics, perceptions seem to be country-specific, however without substantial change in the general interpretation of the data. It would be interesting to further explore which characteristics (of patients, culture, the health care system, etc.) contribute to these differences across Europe.

Lastly, many of our survey participants provided additional information and explanations as free comments. In the view of the richness and magnitude of these data, we are considering to summarize these individual comments in a representative by means of a future qualitative, thematic analysis.

### Conclusions

This study suggests that part of the gap between desired and observed modality mix of renal replacement therapies in Europa may be due to suboptimal information provision to patients, as well as to patients not being offered the full range of treatment options in an equal and unbiased manner. There was an association between involvement in modality selection and patient satisfaction. Health care providers, researchers and guideline developers should focus on developing and applying tools to facilitate the engagement of patients in modality selection.
